# Induced Marine Fungus *Chondrostereum* sp. as a Means of Producing New Sesquiterpenoids Chondrosterins I and J by Using Glycerol as the Carbon Source

**DOI:** 10.3390/md12010167

**Published:** 2014-01-07

**Authors:** Hou-Jin Li, Wen-Han Jiang, Wan-Ling Liang, Jia-Xin Huang, Yu-Fei Mo, Yan-Qing Ding, Chi-Keung Lam, Xiao-Jun Qian, Xiao-Feng Zhu, Wen-Jian Lan

**Affiliations:** 1 School of Chemistry and Chemical Engineering, Sun Yat-Sen University, Guangzhou 510275, China; E-Mails: ceslhj@mail.sysu.edu.cn (H.-J.L.); natprodjwh@gmail.com (W.-H.J.); natprodhjx1@gmail.com (J.-X.H.); cklam@mail.sysu.edu.cn (C.-K.L.); 2 School of Pharmaceutical Sciences, Sun Yat-Sen University, Guangzhou 510006, China; E-Mails: natprodlwl@gmail.com (W.-L.L.); marinemyf@yahoo.com (Y.-F.M.); marinedyq@gmail.com (Y.-Q.D.); 3 State Key Laboratory of Oncology in South China, Cancer Center, Sun Yat-Sen University, Guangzhou 510060, China; E-Mails: marineqxj@gmail.com (X.-J.Q.); zhuxfeng@mail.sysu.edu.cn (X.-F.Z.); 4 Guangdong Technology Research Center for Advanced Chinese Medicine, Guangzhou 510006, China

**Keywords:** marine fungus, *Chondrostereum* sp., sesquiterpenoids, chondrosterins, cytotoxic activity

## Abstract

*Chondrostereum* sp., a marine fungus isolated from a soft coral *Sarcophyton tortuosum*, can yield hirsutane framework sesquiterpenoids. However, the metabolites profiles vary dramatically with the composition change of the culture media. This fungus was cultured in a liquid medium containing glycerol as the carbon source, and two new metabolites, chondrosterins I and J (**1** and **2**), were obtained. Their structures were elucidated primarily based on MS, NMR and X-ray single-crystal diffraction data. By comparison with the known hirsutane sesquiterpenoids, chondrosterins I and J have unique structural features, including a methyl was migrated from C-2 to C-6, and the methyl at C-3 was carboxylated. Compound **2** exhibited potent cytotoxic activities against the cancer cell lines CNE-1 and CNE-2 with the IC_50_ values of 1.32 and 0.56 μM.

## 1. Introduction

The previous works on the metabolites of marine fungus *Chondrostereum* sp. afforded a series of new hirsutane sesquiterpenoids, including chondrosterins A (**3**)–F and hirsutanol E, together with the known compounds hirsutanols A (**4**), C, F, arthrosporone and incarnal (**5**) [1,2,3]. Chondrosterin A, hirsutanol A, and incarnal exhibited potent cytotoxic activities against various cancer cell lines. The anticancer molecular mechanisms investigation showed that hirsutanol A could induce apoptosis and autophagy via accumulation of reactive oxygen species (ROS) [4,5,6].

**Figure 1:**
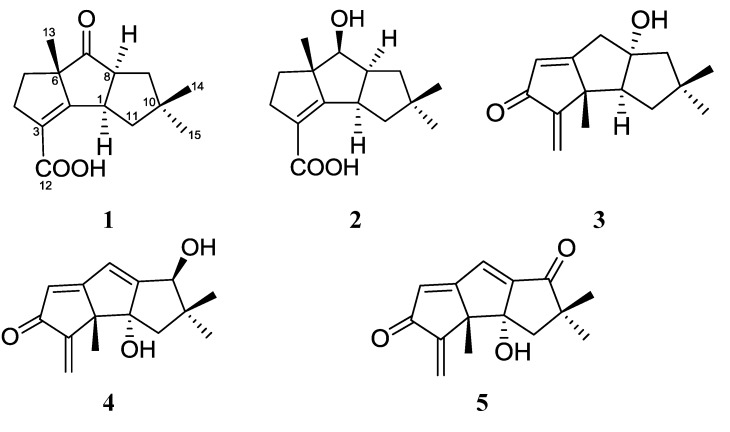
Chemical structures of compounds **1**–**5**.

*Chondrostereum* sp. was previously cultured in glucose-peptone-yeast (GPY) and potato-dextrose (PD) media, containing glucose as the carbon source. Recently, the fungus grew in a GPY media, which using glycerol as the carbon source instead of glucose. The metabolite profiles were analyzed by HPLC, and the result showed that the constituent and content of the metabolites extract of glycerol-containing culture were distinct different from those previous glucose containing media (Supplementary Figure S1). Furthermore, the ethyl acetate extract of the culture broth showed significant cytotoxic activity. These preliminary findings suggest that fungus *Chondrostereum* sp. grown in different carbon source media may produce more novel bioactive metabolites. The metabolite isolation of the marine fungus cultured in glycerol-containing media afforded two new sesquiterpenoids chondrosterins I and J (**1** and **2**, Figure 1). Herein, we report their isolation, structure elucidation, and cytotoxic activities evaluation.

## 2. Results and Discussion

**Figure 2:**
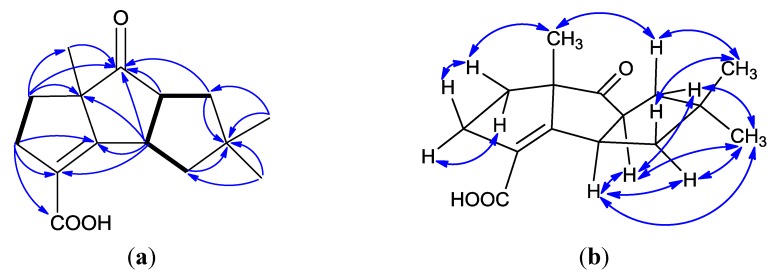
(**a**) ^1^H–^1^H COSY correlations (bold lines), the main HMBC correlations (arrows); and (**b**) key NOESY correlations for **1**.

Chondrosterin I (**1**) was obtained as colorless solid. The molecular formula of **1** was established as C_15_H_20_O_3_ based on the HREIMS peak at *m/z* 248.1407 [M]^+^ (Supplementary Figures S2 and S3) and ^13^C NMR data (Table 1). In the IR spectrum, the prominent bands at 1736 and 1673 cm^−^^1^ indicated the presence of ketone carbonyl and carboxyl groups. The ^13^C NMR and DEPT spectra displayed three methyls, four methylenes, two methines and six quaternary carbons (Supplementary Figures S4–S6). One carbonyl carbon (δ_C_ 220.1), one carboxyl group (δ_C_ 170.1), and one tetrasubstituted double bond (δ_C_ 169.9 and 126.6), represented three double bond equivalents. Thus, **1** must be tricyclic to account for the six double bond equivalents required by the molecular formula. The UV maximum absorption at 244 nm indicated the tetrasubstituted double bond connected to the carboxyl group and formed a conjugated system. The cross-peaks of H-1/H-8, H-1/H-11, H-8/H-9 and H-4/H-5 in ^1^H–^1^H COSY showed the partial structures –CH_2_CHCHCH_2_– and –CH_2_CH_2_– in this molecule (Figure 2a, and Supplementary Figure S7). Three methyl groups with singlets were connected to quaternary carbons. Based on their HMBC correlations, two methyl groups with singlets at δ_H_ 1.00 and 1.14 were connected to quaternary carbon C-10 (δ_C_ 43.5), the other methyl group with singlet at δ_H_ 1.44 was connected to C-6 (δ_C_ 63.8). The HMBC correlations of H-1/C-2, H-1/C-8, H-4/C-3, H-4/C-12, H-5/C-6, H-8/C-7, H-9/C-10, H-13/C-7 established the planar structure of compound **1** (Supplementary Figure S8). The NOESY correlations of H-1/H-8, H-1/H-11α (δ_H_ 2.03), H-1/H-15 (δ_H_ 1.00), H-8/H-9α (δ_H_ 2.03), H-8/H-15 (Figure 2b) established all these protons as α-oriented. No NOESY correlation was observed between H-1/H-13 and H-8/H-13 indicated the methyl group (H-13) was placed at the β-oriented (Supplementary Figure S9).

**Table 1:** ^1^H and ^13^C NMR data for compound **1** obtained at 400/100 MHz, in CDCl_3_.

Position	δ_C_, Type	δ_H_, Mult., ( *J*)
1	42.2, CH	3.96, ddd (9.6, 9.6, 9.6)
2	169.9, C	
3	126.6, C	
4	32.5, CH_2_	α: 2.89, ddd (16.0, 10.4, 6.4); β: 2.72, dd (16.0, 8.8)
5	35.6, CH_2_	α: 1.99, m; β: 1.85, m
6	63.8, C	
7	220.1, C	
8	58.2, CH	3.22, ddd (9.6, 9.6, 9.6)
9	46.1, CH_2_	α: 2.03, m; β: 1.68, m
10	43.5, C	
11	46.1, CH_2_	α: 2.03, m; β: 1.59, m
12	170.1, C	
13	23.8, CH_3_	1.44, s
14	28.2, CH_3_	1.14, s
15	26.5, CH_3_	1.00, s
12-OH		10.50, brs

**Figure 3:**
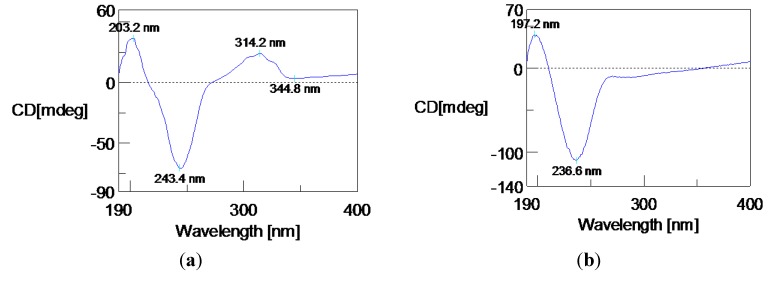
Circular dichroism (CD) spectra of compounds **1** (**a**) and **2** (**b**).

**Figure 4:**
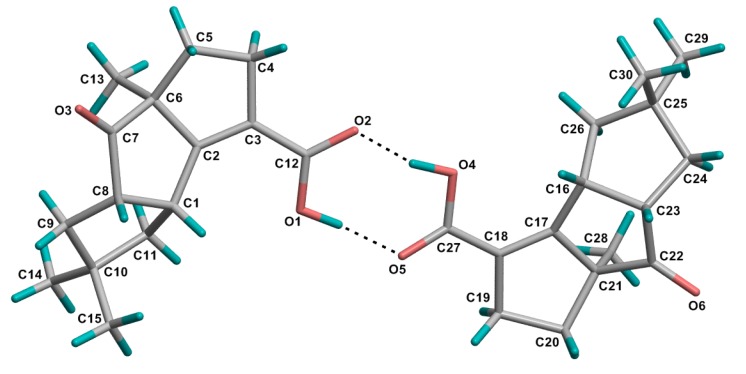
Crystal structure of **1**. Thermal ellipsoids are plotted at 30% probability level.

Circular dichroism (CD) spectrum of **1** exhibited a positive Cotton effect (Figure 3a). Having a cyclopentanone skeleton in this molecule, the octant rule was allowed to assign the absolute configuration. The absorption peak at 314.2 nm was due to an *n*-π* transition of the ketone group. The C-9, C-10, C-11, C-14 and C-15 resided in rear upper left and C-3, C-4, C-5 and C-12 located in rear lower right octants made significant positive contributions to the Cotton effect. So, the absolute configuration 1*R*, 6*S*, 8*S* was determined. Finally, the structure of **1**, and the absolute configuration 1*R*, 6*S* and 8*S* were confirmed by X-ray single-crystal diffraction data (Figure 4). The asymmetric unit contains two crystallographically independent molecules, which are joined together by a strong pair of O-H...O=C hydrogen bonds and the crystal structure is further consolidated by weak C-H...O=C intermolecular interactions.

**Table 2:** ^1^H and ^13^C NMR data for compound **2** obtained at 400/100 MHz.

Position	δ_C_, Type ^a^	δ_H_, Mult., (*J*) ^a^	δ_C_^b^	δ_H_, Mult., (*J*) ^b^
1	42.1, CH	3.55, ddd (9.6, 9.6, 9.6)	42.6	3.53, ddd (9.6, 9.6, 9.2)
2	175.6, C		173.7	
3	123.4, C		124.5	
4	33.8, CH_2_	α: 2.86, ddd (15.6, 9.6, 6.4); β: 2.71, dd (15.6, 6.0)	34.9	α: 2.80, ddd (15.2, 10.8, 6.8); β: 2.62, dd (15.2, 8.0)
5	41.2, CH_2_	α: 1.84, m; β: 1.75, m	42.3	α: 1.75, m; β: 1.70, m
6	61.4, C		62.1	
7	79.0, CH	4.05, d (9.6)	79.1	3.99, d (9.2)
8	51.9, CH	3.21, dddd (9.6, 9.6, 9.6, 6.8)	53.0	3.17, dddd (9.2, 9.2, 9.2,9.2)
9	42.1, CH_2_	α: 1.81, dd (12.0, 6.8); β: 1.48, ddd (12.0, 9.6, 2.0)	43.3	α: 1.97, dd (12.0, 9.2); β: 1.42, ddd (12.0, 9.2, 2.0)
10	41.9, C		42.2	
11	46.6, CH_2_	α: 2.00, ddd (12.8, 9.6, 2.0); β: 1.38, dd (12.8, 9.6)	47.6	α: 1.92, ddd (12.8, 9.6, 2.0); β: 1.38, dd (12.8, 9.6)
12	170.6, C		166.7	
13	18.5, CH_3_	1.25, s	19.2	1.24, s
14	28.7, CH_3_	1.11, s	29.3	1.09, s
15	26.6, CH_3_	0.97, s	27.2	0.95, s
7-OH		4.92, brs		3.84, brs
12-OH		10.53, brs		10.47, brs

^a^ Measured in CDCl_3_, and CDCl_3_ was used as an internal standard (δ_C_ 77.0, δ_H_ 7.26); ^b^ Measured in Acetone-*d*_6_, and Acetone-*d*_6_ was used as an internal standard (δ_C_ 29.92, δ_H_ 2.05).

**Figure 5:**
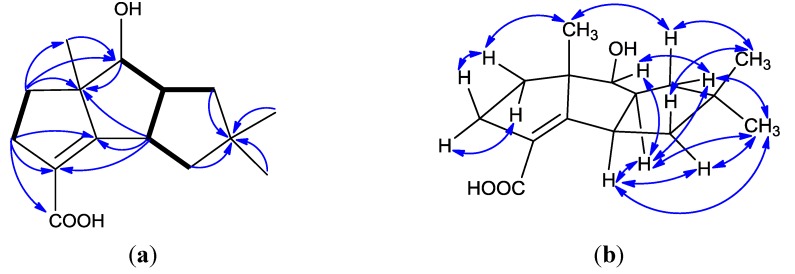
(**a**) ^1^H–^1^H COSY correlations (bold lines), the main HMBC correlations (arrows); and (**b**) key NOESY correlations for **2**.

Chondrosterin J (**2**) was obtained as white solid. The molecular formula of **2** was established as C_15_H_2__2_O_3_ based on the HREIMS peak at *m/z* 250.1563 [M]^+^ and ^13^C NMR data (Table 2, Supplementary Figures S10–S13). The two broad singlets at δ_H_ 4.92 and 10.53 indicated there were two hydroxyl groups in this molecule. The ^13^C NMR and DEPT spectra displayed three methyls, four methylenes, three methines and five quaternary carbons. One carboxyl group (δ_C_ 170.6), and one tetrasubstituted double bond (δ_C_ 175.6 and 123.4), represented two degrees of unsaturation. Thus, **2** must be tricyclic to account for the five degrees of unsaturation required by the molecular formula. By comprehensive analysis, the ^1^H, ^13^C and 2D (HMQC, HMBC, ^1^H-^1^H COSY) NMR data (Figure 5a, Supplementary Figures S14–S16), **2** was identified as the keto reduction product of **1**. In NOESY spectrum, the cross peaks of H-1/H-8, H-1/H-11α (δ_H_ 2.00), H-1/H-15 (δ_H_ 0.97), H-7/H-9α (δ_H_ 1.81), H-8/H-9α, H-8/H-15 (δ_H_ 0.97), H-9α /H-15, and H-11α/H-15 revealed these protons were α-oriented (Figure 5b, and Supplementary Figure S17). No cross peak was observed between H-7 and H-13 (Me) also supported that H-7 and H-13 were α and β-oriented, respectively. CD curve of **2** was similar with **1**, except the absorption peak at 314.2 nm (Figure 3b). Based on the absolute configuration determination of **1**, the absolute configuration of **2** was established as 1*R*, 6*S*, 7*S*, 8*S*.

The human nasopharyngeal cancer cell line CNE-1 and CNE-2 were used to examine the cytotoxic activities of **1** and **2** *in vitro*. Compound **2** showed potent cytotoxic activities against CNE-1 and CNE-2 cell lines with the IC_50_ values of 1.32 and 0.56 μM, respectively. By comparison, compound **2** displayed stronger cytotoxic activities than chondrosterin A (CNE-2: 4.95 μM), hirsutanol A (CNE-1: 10.08 μM; CNE-2: 12.72 μM), and incarnal (CNE-1: 34.13 μM; CNE-2: 24.87 μM). In contrast, **1** was inactive in this assay (IC_50_ values >200 μM).

## 3. Experimental Section

### 3.1. General Experimental Procedures

Preparative HPLC was performed using a Shimadzu LC-20AT HPLC pump equipped with an SPD-20A dual λ absorbance detector and a Shim-pack PRC-ODS HPLC column (250 × 20 mm). Optical rotations were measured using a Schmidt and Haensch Polartronic HNQW5 optical rotation spectrometer. CD spectra were measured on a JASCO J-810 circular dichroism spectrometer. IR spectra were recorded on a PerkinElmer Frontier FT-IR spectrophotometer. UV spectra were recorded on a Shimadzu UV-Vis-NIR spectrophotometer. 1D and 2D NMR spectra were recorded on a Bruker Avance II 400 spectrometer. The chemical shifts are relative to the residual solvent signals (CDCl_3_: δ_H_ 7.26 and δ_C_ 77.0; Acetone-*d*_6_: δ_H_ 2.05 and δ_C_ 29.92). Mass spectra were obtained on a Thermo DSQ EI low-resolution mass spectrometer and a Thermo MAT95XP EI high-resolution mass spectrometer. X-ray diffraction data were acquired on a Bruker SMART APEX CCD X-ray single crystal diffractometer.

### 3.2. Fungal Strain and Culture Method

*Chondrostereum* sp. was isolated from the inner tissue of a soft coral of the species *Sarcophyton tortuosum* collected from the Hainan Sanya National Coral Reef Reserve, China. This fungal strain was maintained in sterile aqueous solution of 15% (v/v) glycerol at −80 °C. The fermentation medium was glycerol 10 g, peptone 5 g, yeast extract 2 g, CaCO_3_ 1 g, seawater 1 L. The pH of the medium was adjusted to 7.5 by adding either HCl (1 N) or NaOH (30%, w/v) solution. The mycelia were aseptically transferred to 500 mL Erlenmeyer flasks containing 200 mL of the sterile liquid medium. The flasks were then incubated at 28 °C on a rotary shaker (120 rpm) for 20 days.

### 3.3. Extraction and Isolation

Sixty liters of liquid culture was filtered through cheesecloth. The culture broth was successively extracted three times with EtOAc. The EtOAc extract was concentrated by low-temperature rotary evaporation. The extract (24.6 g) was chromatographed on a silica gel column using petroleum ether–EtOAc (100:0–0:100) followed by EtOAc–MeOH (100:0–0:100) as the eluent to afford 12 fractions (code Fr. 1–Fr. 12). Fr. 4 was further purified by RP-HPLC with a gradient of H_2_O–MeCN (40:60 up to 0:100, v/v) to afford compounds **1** (21 mg) and **2** (12 mg).

Chondrosterin I (**1**): Colorless solid; 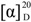 −17.73 (*c* 0.015, MeOH); UV (MeOH) λ_max_ (ε) 244 nm (9310), 219 nm (7344); IR (KBr) υ_max_ 2956, 2920, 2851, 1736, 1673, 1648, 1464, 1434, 1371, 1334, 1281, 1266, 1209, 1119, 1077, 927, 905, 763, 719, 711 cm^−^^1^; ^1^H and ^13^C NMR data, see Table 1; LREIMS *m*/*z* 248, 233, 220, 204, 187, 174, 159, 147, 132, 119, 105, 77, 65, 55; HREIMS *m*/*z* 248.1406 [M]^+^ (calcd for C_15_H_20_O_3_, 248.1407).

Chondrosterin J (**2**): White solid; 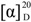 −9.11 (*c* 0.006, MeOH); UV (MeOH) λ_max_ (ε) 237 nm (14829); IR (KBr) υ_max_ 3372, 2952, 2924, 2866,1677, 1463, 1445, 1422, 1385, 1366, 1329, 1279, 1263, 1245, 1228, 1197, 1158, 1135, 1100, 1060, 1030, 1015, 971, 912, 811, 764, 691, 665 cm^−1^; ^1^H and ^13^C NMR data, see Table 2. LREIMS *m*/*z* 250, 232, 214, 204, 199, 187, 171, 145, 131, 119, 105, 91, 77, 69, 55; HREIMS *m*/*z* 250.1563 [M]^+^ (calcd for C_15_H_22_O_3_, 250.1563).

### 3.4. Crystal Structure Determination of **1**

Crystals of **1** was obtained from EtOAc solution. Chondrosterin I (**1**): C_15_H_20_O_3_, *M* = 248.31, colourless block, orthorhombic system, space group *P*2_1_2_1_2_1_, *a* = 6.4797(2), *b* = 8.3776(2), *c* = 50.4166(14) Å, *V* = 2736.83(13) Å^3^, *Z* = 4, *d* = 1.205 g/cm^3^, crystal size 0.40 × 0.38 × 0.36 mm^3^. The flack parameter is 0.1648. X-ray diffraction data were collected on a Bruker SMART APEX CCD diffractometer with Cu *K_α_* radiation (λ = 1.54178Å) at a temperature of 173 K. The data were processed using CrysAlis. The structures were solved by direct method. H-atoms were added in ideal positions and refined as riding models. The structures were refined using full-matrix least-squares based on *F*^2^ with program SHELXL [7,8].

CCDC967610 contains the supplementary crystallographic data of compound **1** [9].

### 3.5. Cytotoxicity Assay

The *in vitro* cytotoxicities of **1** and **2** were determined using the colorimetric MTT (3-(4,5-dimethylthiazol-2-yl)-2,5-diphenyl-2*H*-tetrazolium bromide) assay. The tested human nasopharyngeal carcinoma cell lines CNE-1 and CNE-2 were seeded in 96-well plates at a density of 3 × 10^7^ cells/L, and the compounds were added at various concentrations (0.0125–5 μg/mL). After 48 h, MTT was added to the culture medium at a final concentration of 0.5 mg/mL, and the plates were incubated for 4 h at 37 °C. The supernatant was removed. The formazan crystals were dissolved in DMSO (150 μL) with gentle shaking at r.t. The absorbance at 570 nm was recorded with a microplate reader (Bio-Rad, Hercules, California, CA, USA), and the data were analyzed with the SPSS 13.0 software package. Hirsutanol A was used as a positive control and it showed cytotoxic activities against CNE-1 and CNE-2 cell lines with the IC_50_ values of 10.08 and 12.72 μM, respectively.

## 4. Conclusions

Our results indicated that the marine fungus *Chondrostereum* sp. could produce novel metabolites with various structures. Hopefully, the systematical altering the component of the culture media can more fully explore the biosynthesis potential of this fungus. Compounds **1** and **2** belong to hirsutane sesquiterpenoids, and have some unique features, including a rearranged hirsutane skeleton which may be derived by migration of a methyl group from C-2 to C-6, a double bond between C-2 and C-3, and a carboxyl group connected to C-3. To the best of our knowledge, the hirsutane sesquiterpenoids, complicatic acid [10], pleurotellic acid [11], phellodonic acid [12], chlorostereone [13] and hirsutic acid [14], have a carboxyl group in each molecule. However, the carboxyl group placed at C-3 is unprecedented.

## Supplementary Files

Supplementary File 1 Supplementary Information (PDF, 863 KB)
