# Supplementary material for: Induced Marine Fungus Chondrostereum sp. as a Means of Producing New Sesquiterpenoids Chondrosterins I and J by Using Glycerol as the Carbon Source
Source: Mar Drugs. 2014 Jan 7;12(1):167–75. doi: 10.3390/md12010167 (PMC3917267; doi:10.3390/md12010167)

Supplementary Information

Figure S1. HPLC analysis on the fungal metabolites in different culture media.
Figure S2. LREIMS of chondrosterin I (**1**).
Figure S3. HREIMS of chondrosterin I (**1**).
Figure S4. ^{1}H NMR (400 MHz, $CDCl_3$) spectrum of chondrosterin I (**1**).
Figure S5. ^{13}C NMR (100 MHz, $CDCl_3$) spectrum of chondrosterin I (**1**).
Figure S6. gHMQC of chondrosterin I (**1**).
Figure S7. ^{1}H-^{1}H gCOSY of chondrosterin I (**1**).
Figure S8. gHMBC of chondrosterin I (**1**).
Figure S9. NOESY of chondrosterin I (**1**).
Figure S10. LREIMS of chondrosterin J (**2**).
Figure S11. HREIMS of chondrosterin J (**2**).
Figure S12. ^{1}H NMR (400 MHz, $CDCl_3$) spectrum of chondrosterin J (**2**).
Figure S13. ^{13}C NMR (100 MHz, $CDCl_3$) spectrum of chondrosterin J (**2**).
Figure S14. gHMQC spectrum of chondrosterin J (**2**).
Figure S15. ^{1}H-^{1}H gCOSY spectrum of chondrosterin J (**2**).
Figure S16. gHMBC spectrum of chondrosterin J (**2**).
Figure S17. NOESY of chondrosterin J (**2**).

Figure S1. HPLC analysis on the fungal metabolites in different culture media.

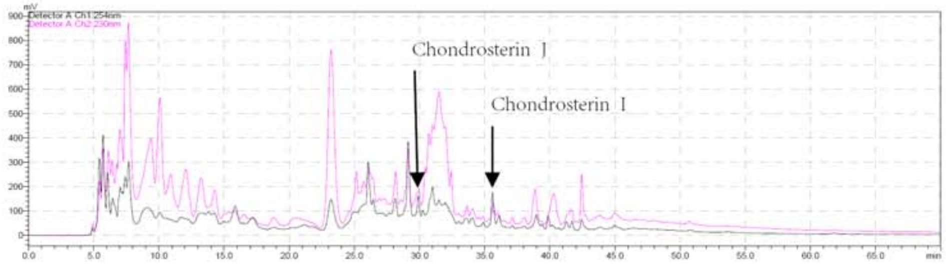

(a)Fermentation medium: glycerol 10 g, peptone 5 g, yeast extract 2 g, $CaCO_3$ 1 g, seawater 1 L

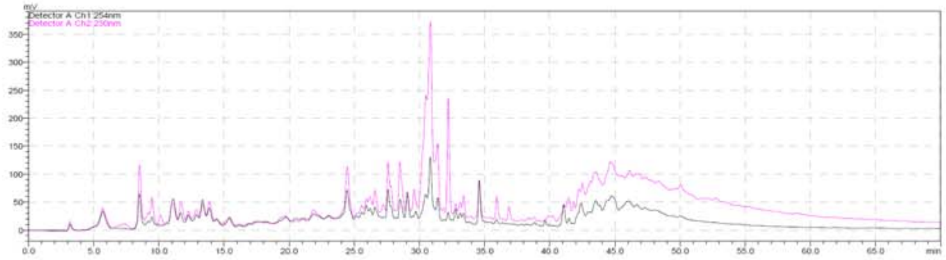

(b) Fermentation medium: glucose 10g/L, peptone 5g/L, yeast extract 2g/L, sea water 1L, pH 7.5.

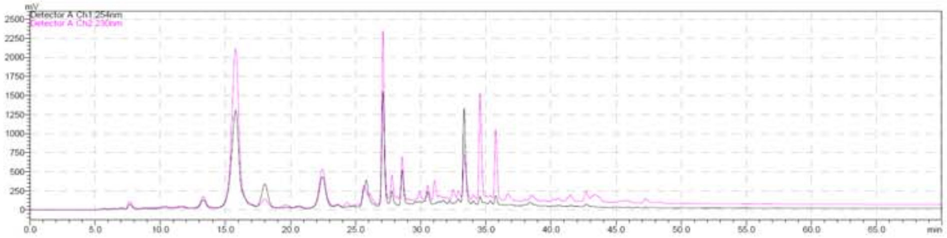

(c) Fermentation medium: potatoes 200 g, dextrose 20 g, seawater 1 L.

Shimadzu LC-20AT HPLC pump equipped with a SPD-20A dual λ absorbance detector and Inertsil® ODS-SP HPLC column (250 × 4.6 mm, 5μm).

LC Time Prog.

Time/min	Module	Action	Value
0.01	Pumps	B.Conc	30
10.00	Pumps	B.Conc	30
40.00	Pumps	B.Conc	100
60.00	Pumps	B.Conc	100
65.00	Pumps	B.Conc	30
70.00	Pumps	B.Conc	30
75.00	Controller	Stop	

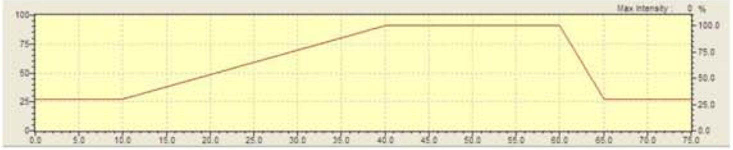

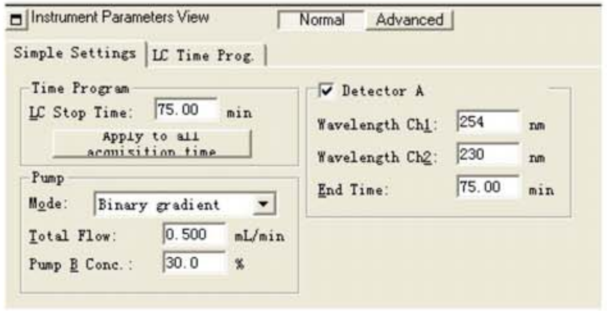

Figure S2. LREIMS of chondrosterin I (**1**).

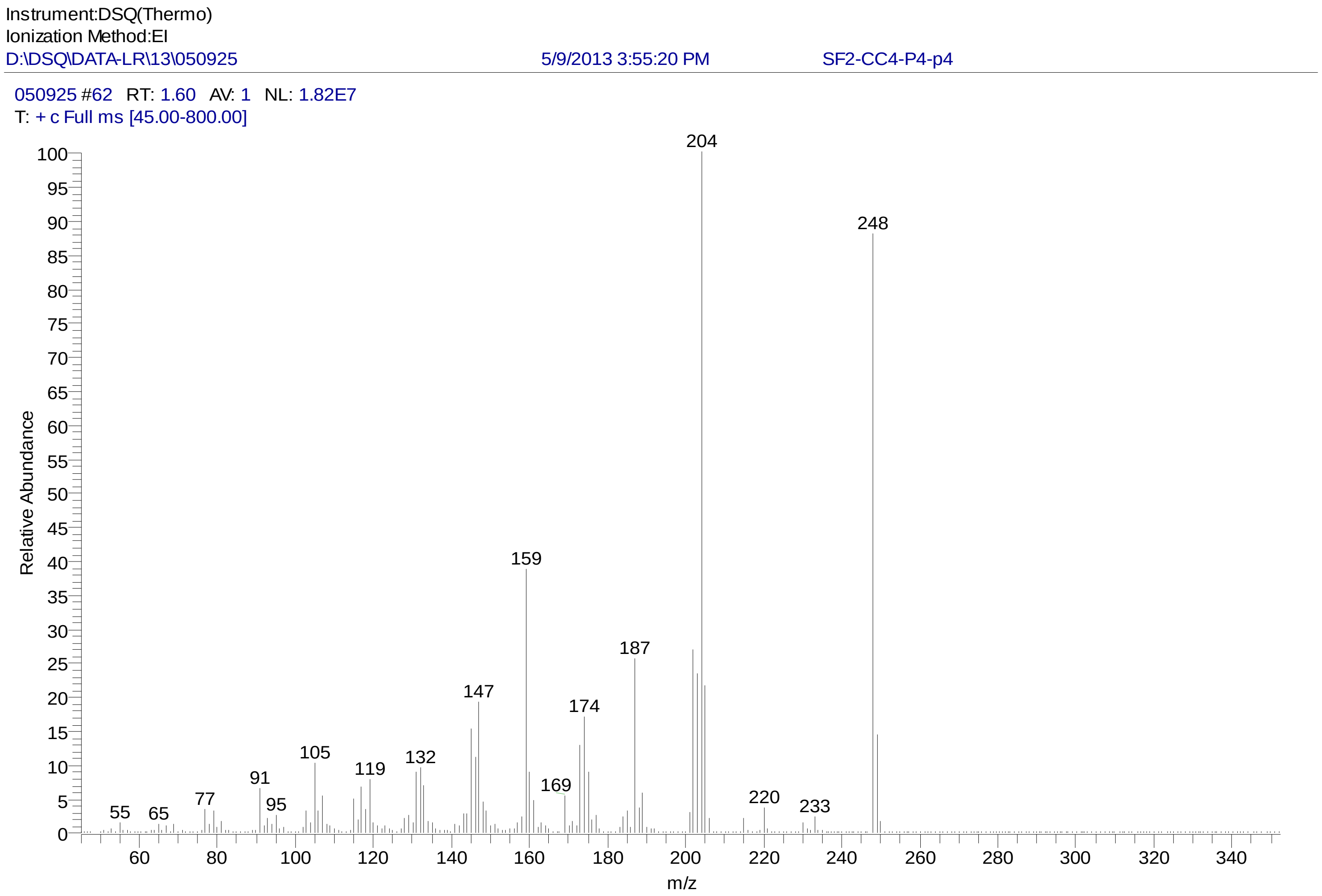

Figure S3. HREIMS of chondrosterin I (**1**).

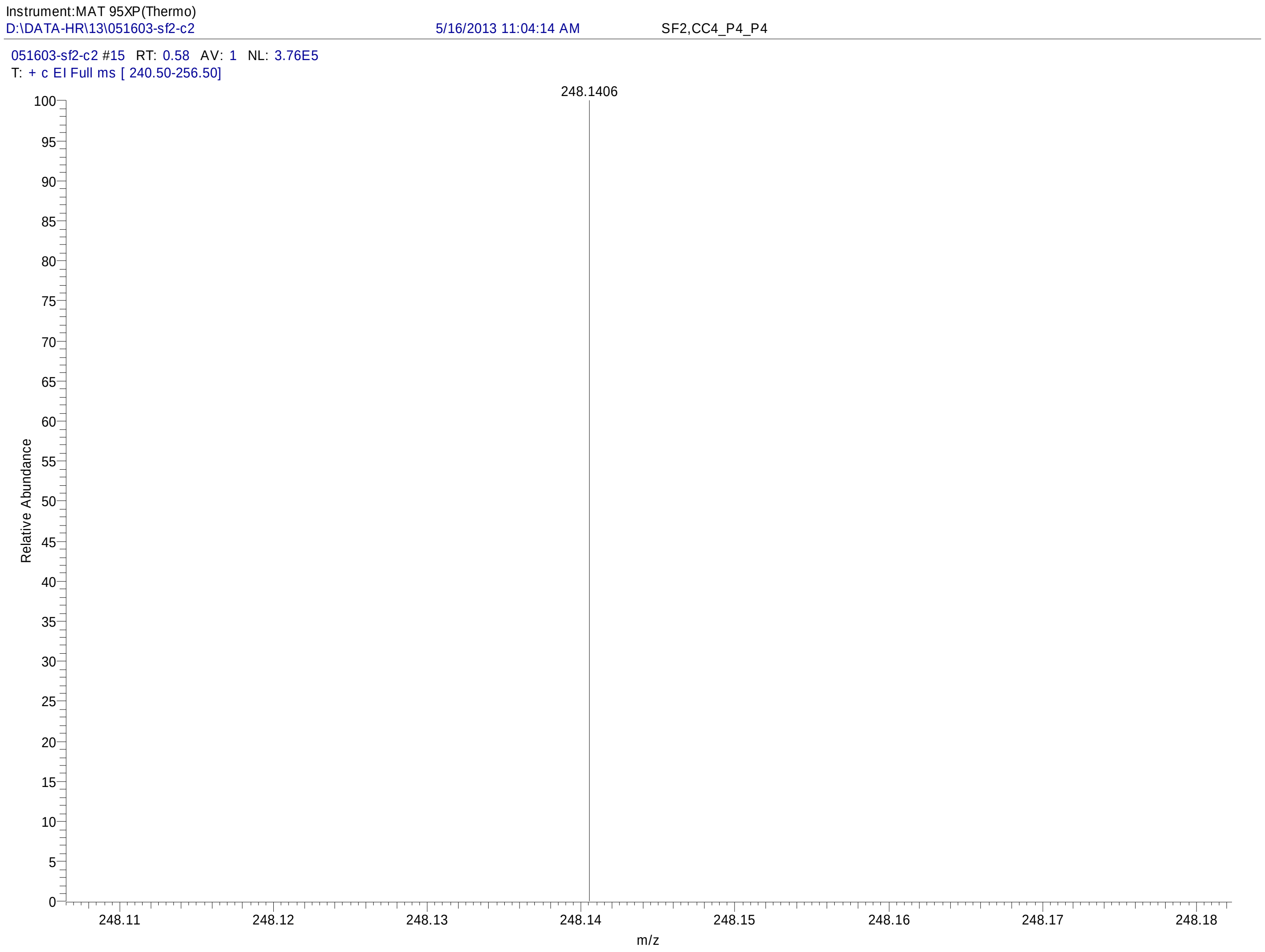

Figure S4. ^{1}H NMR (400 MHz, $CDCl_3$) spectrum of chondrosterin I (**1**).

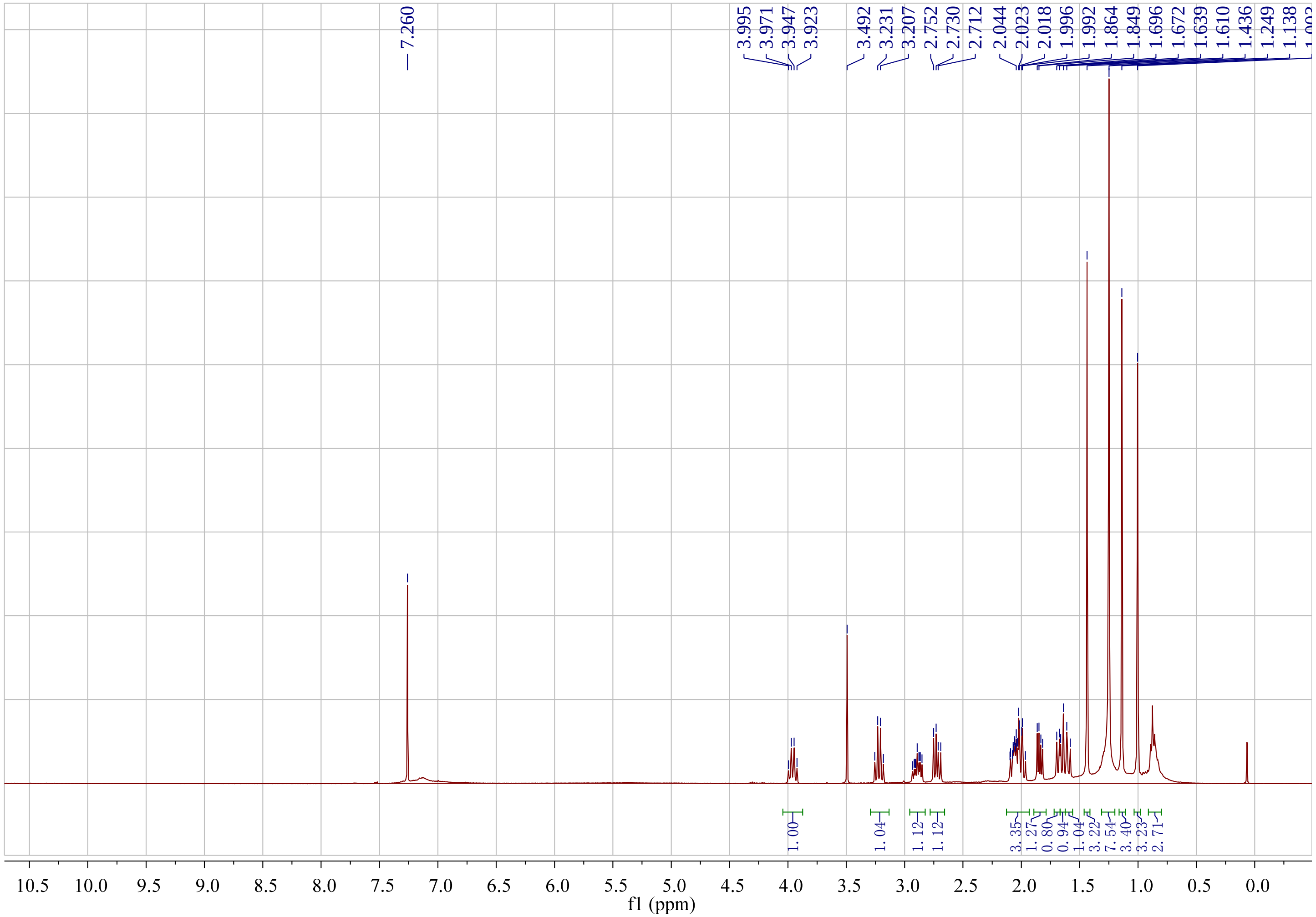

Figure S5. ^{13}C NMR (100 MHz, $CDCl_3$) spectrum of chondrosterin I (**1**).

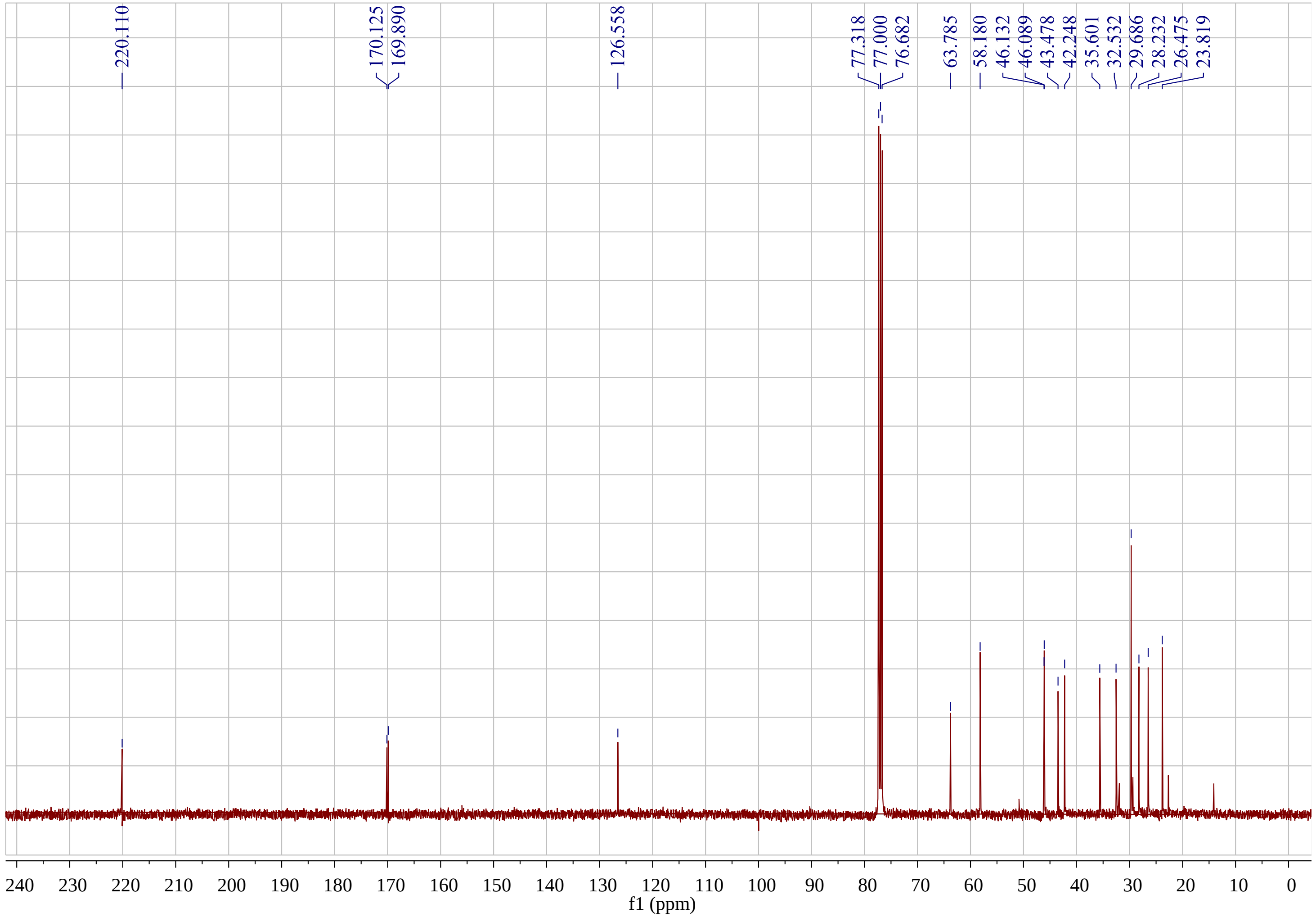

Figure S6. gHMQC of chondrosterin I (**1**).

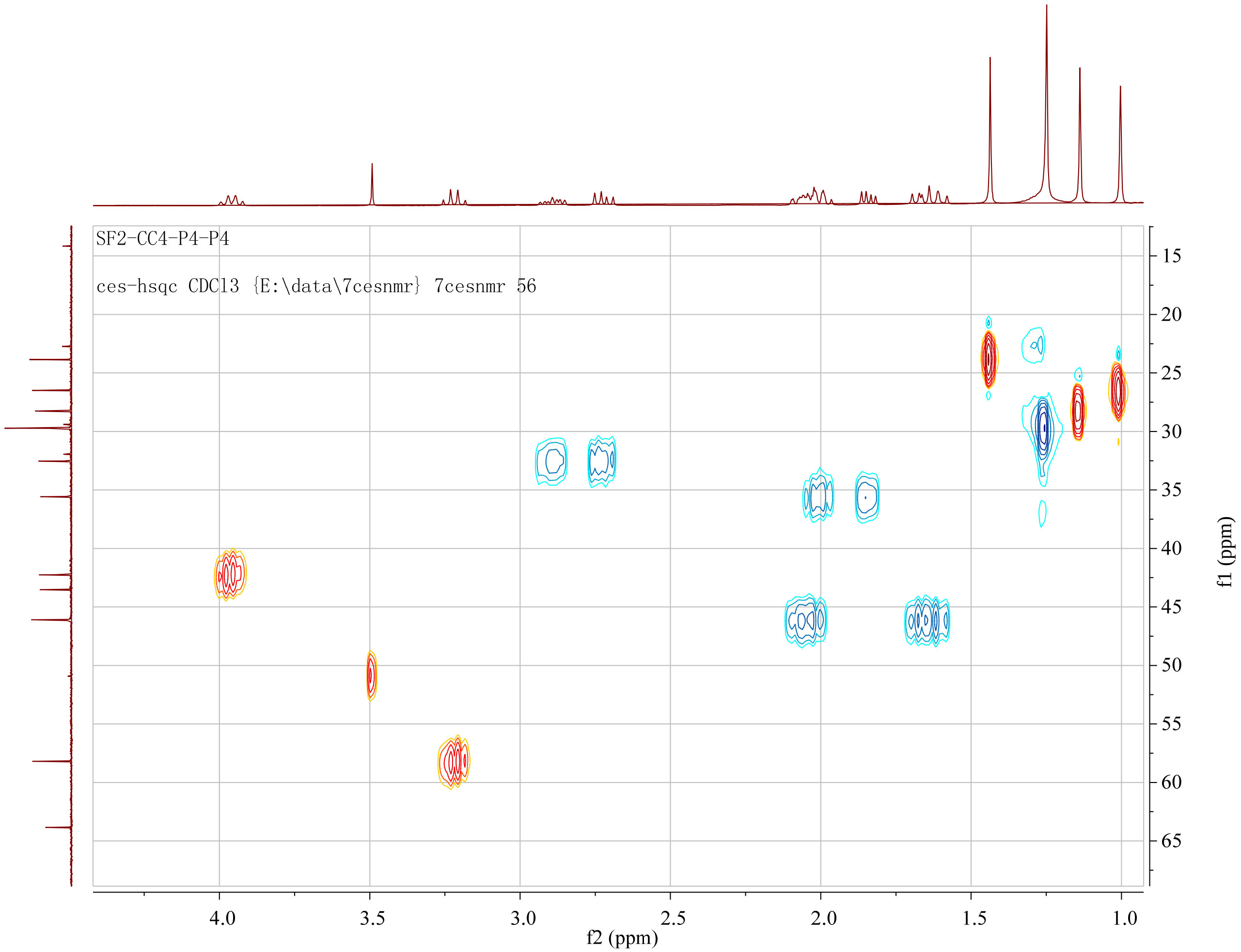

Figure S7. ^{1}H-^{1}H gCOSY of chondrosterin I (**1**).

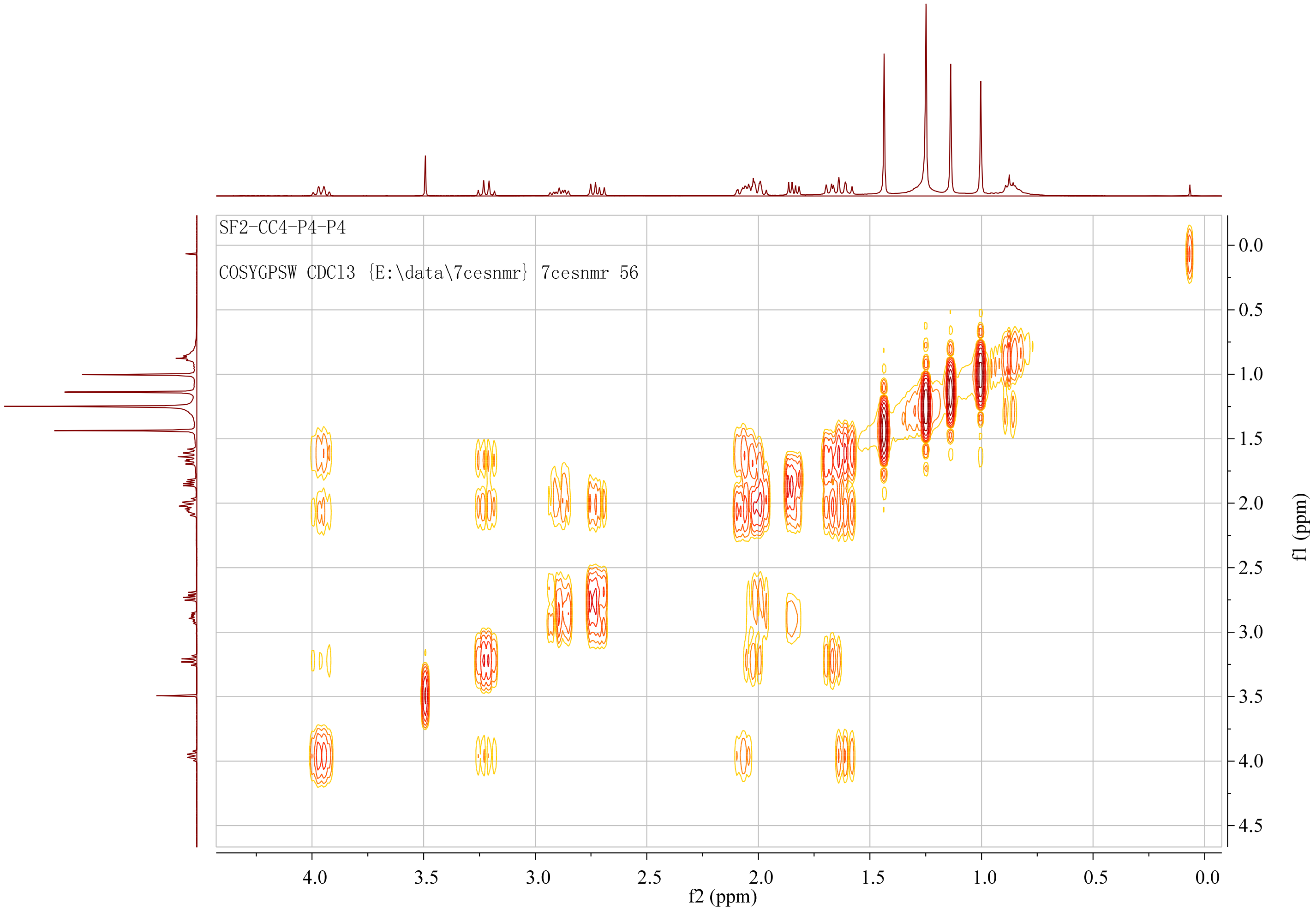

Figure S8. gHMBC of chondrosterin I (**1**).

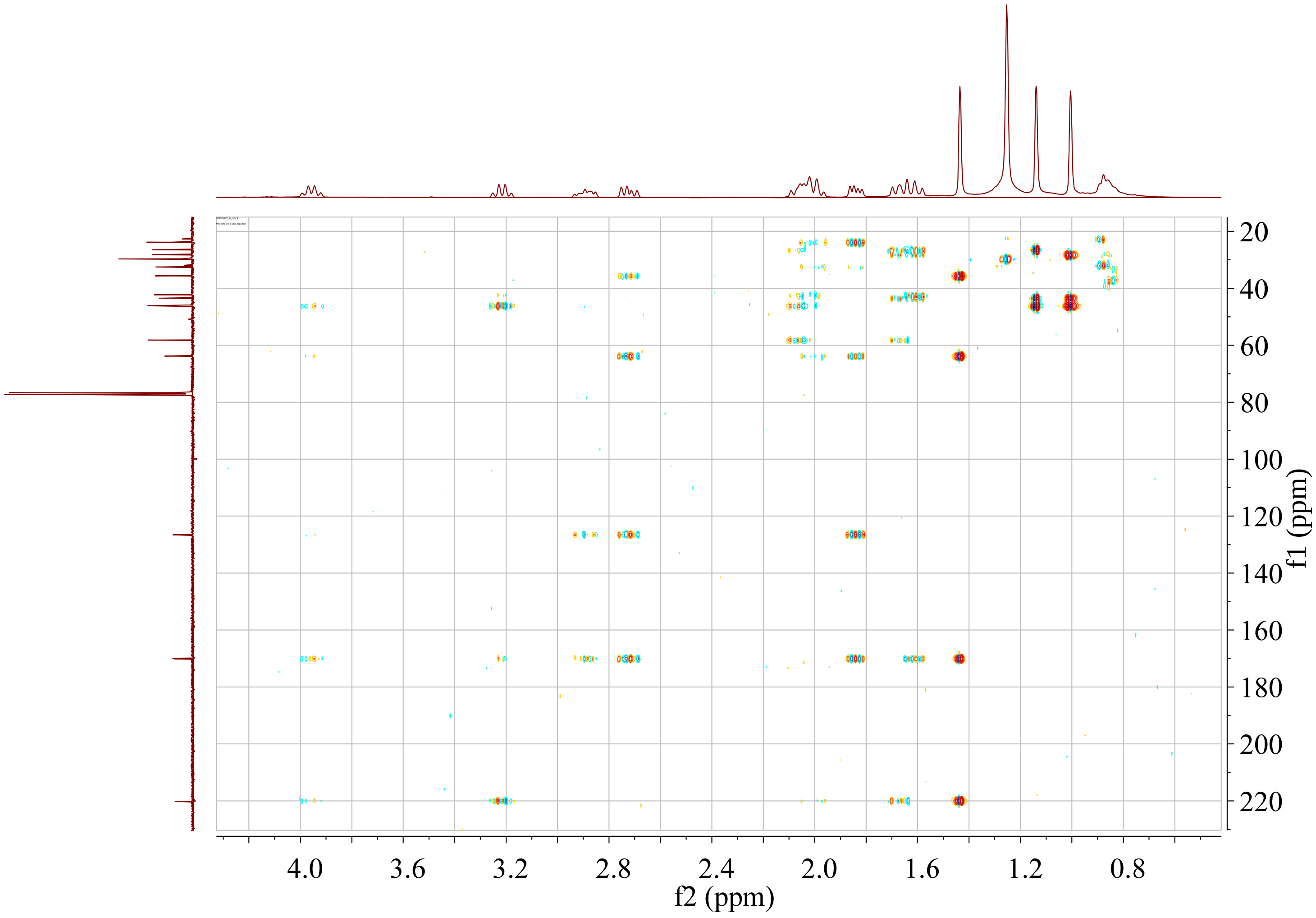

Figure S9. NOESY of chondrosterin I (**1**).

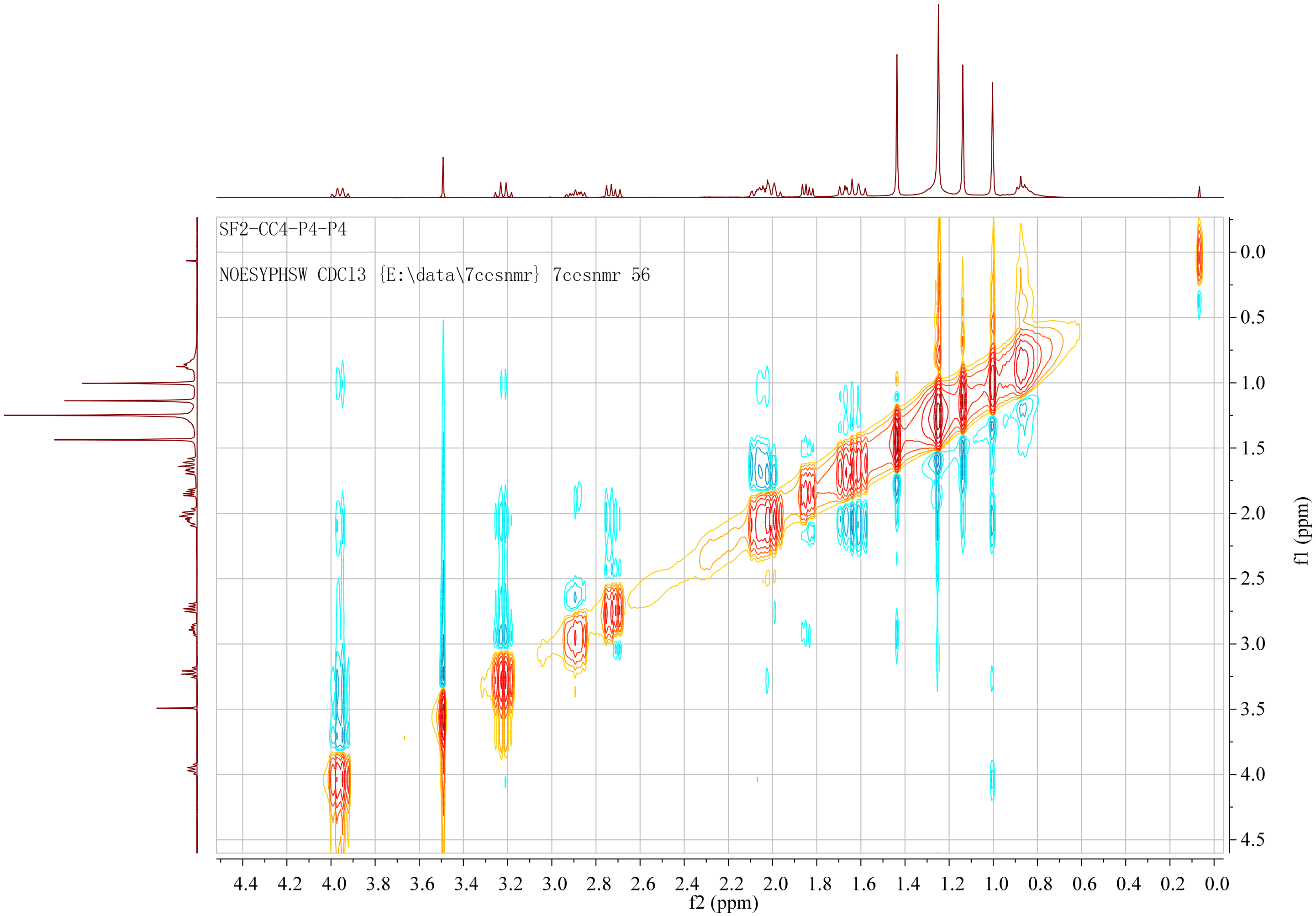

Figure S10. LREIMS of chondrosterin J (**2**).

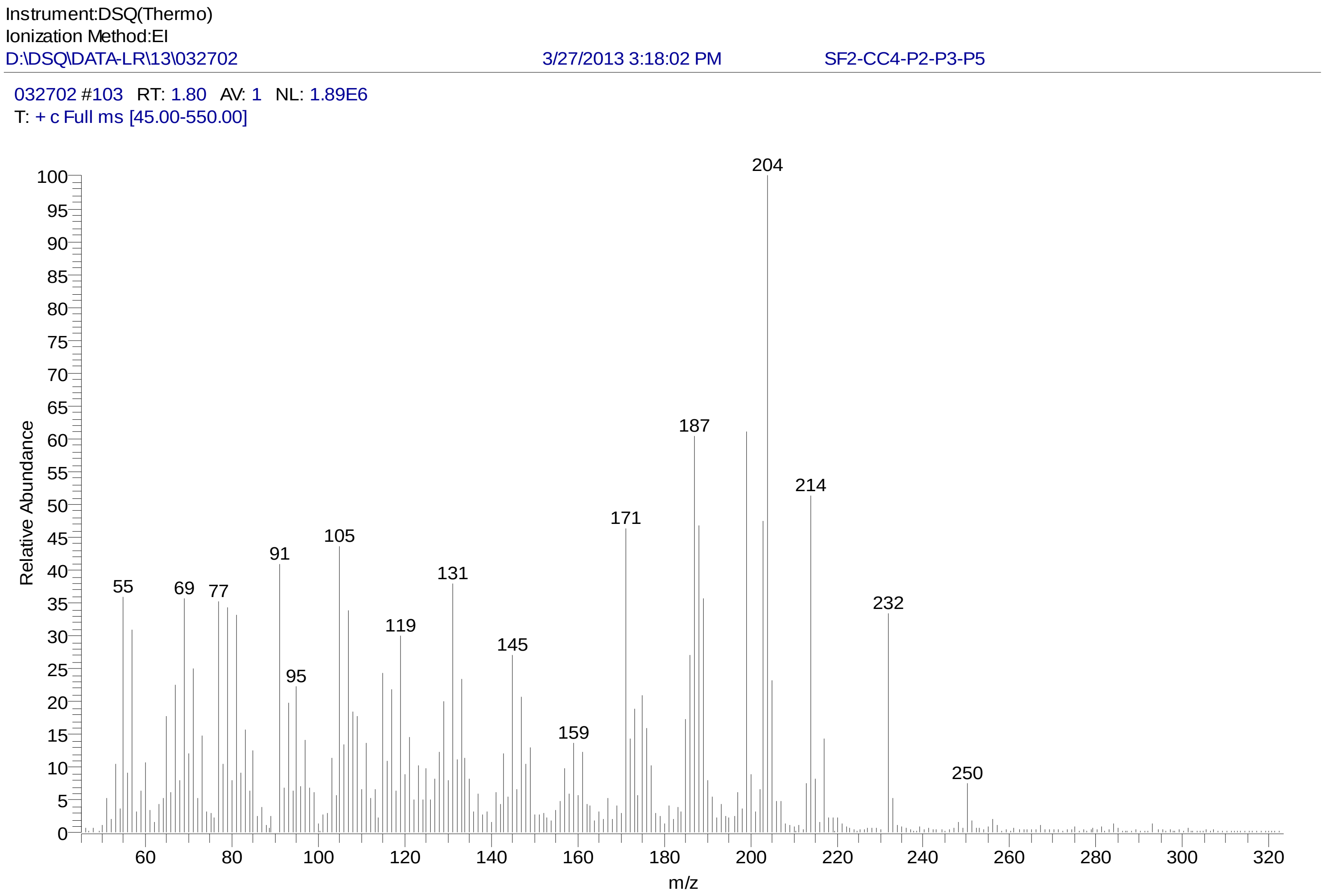

Figure S11. HREIMS of chondrosterin J (**2**).

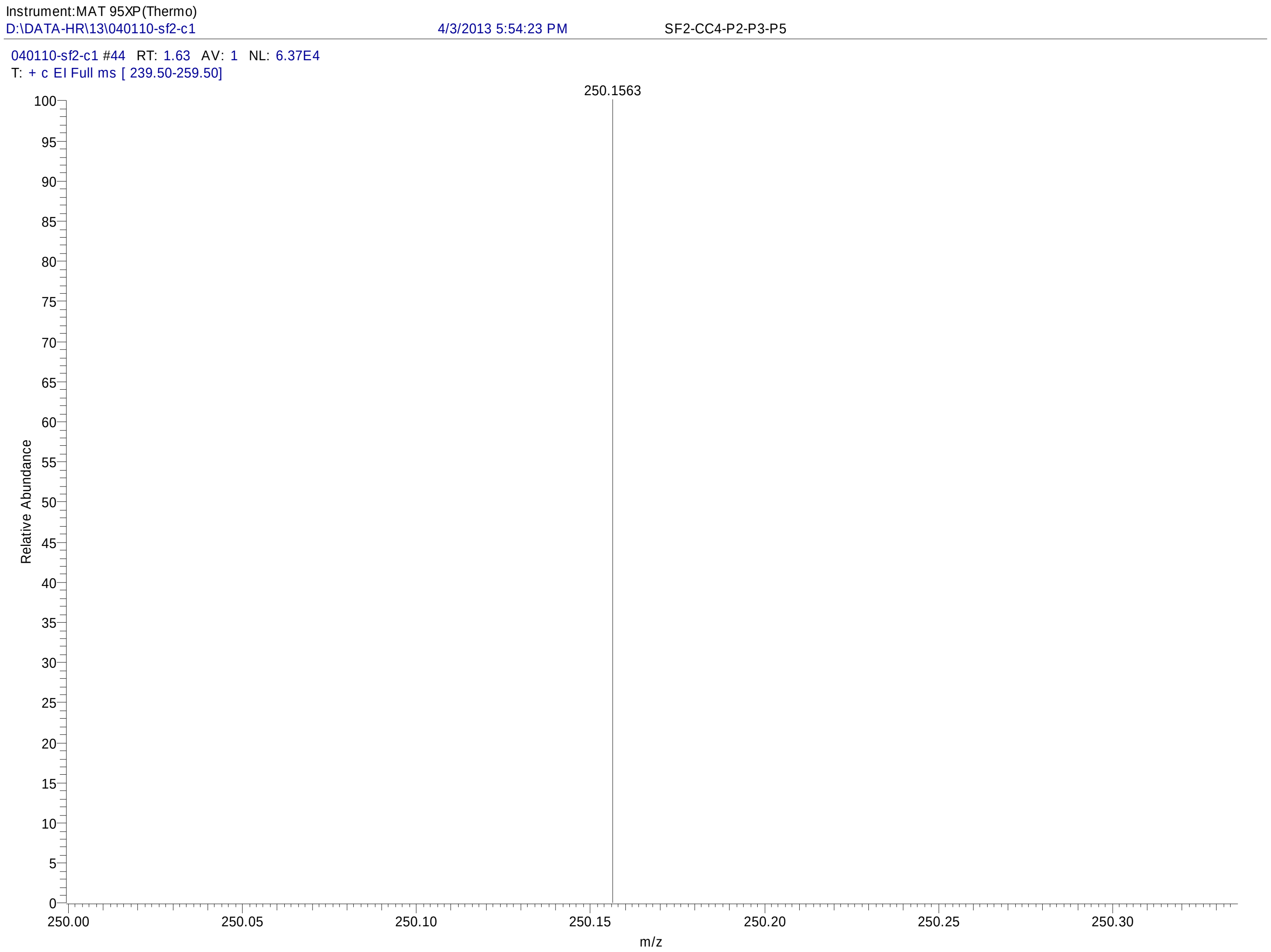

Figure S12. ^1H NMR (400 MHz, $CDCl_3$) spectrum of chondrosterin J (**2**).

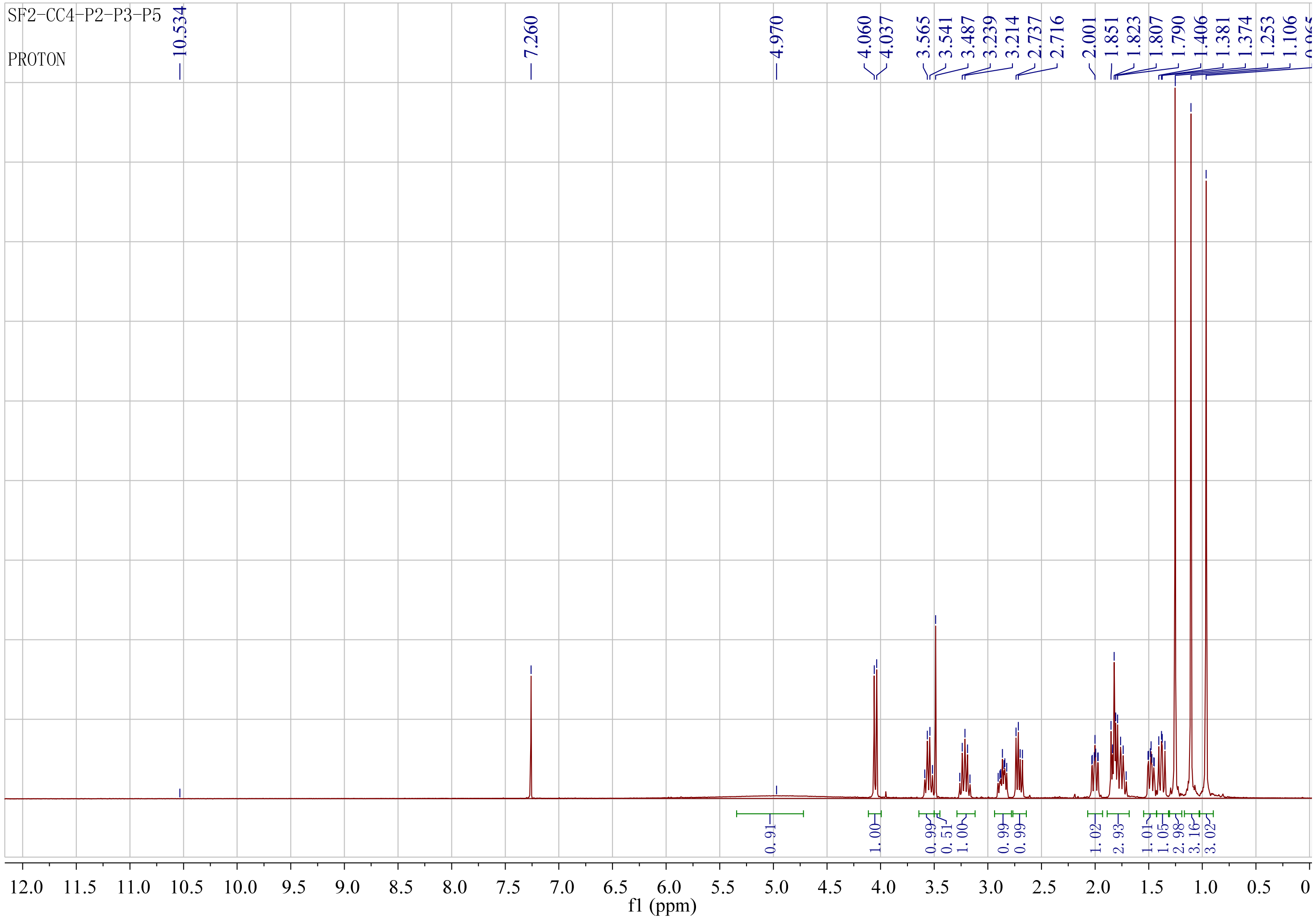

Figure S13. ^{13}C NMR (100 MHz, $CDCl_3$) spectrum of chondrosterin J (**2**).

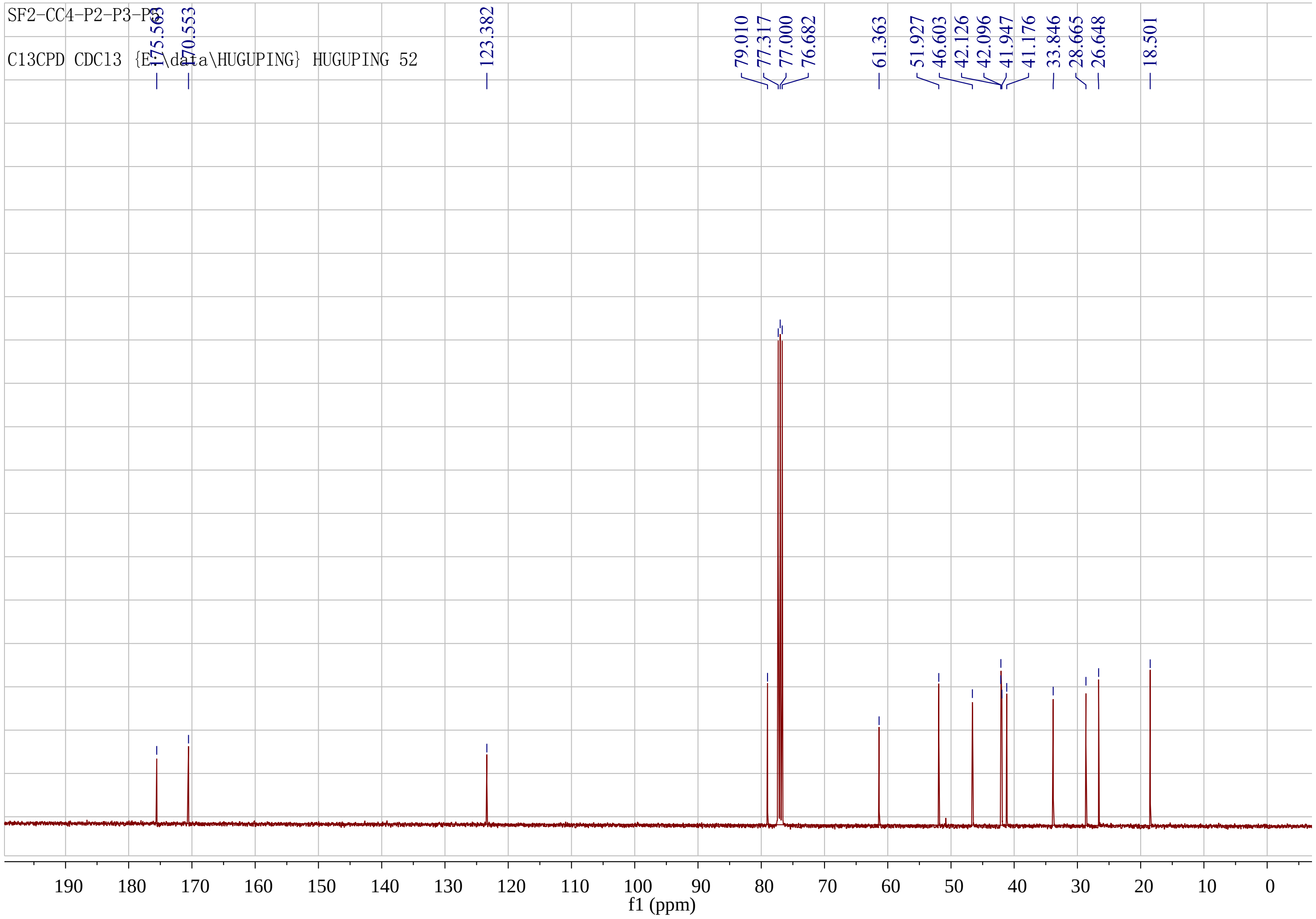

Figure S14. gHMQC spectrum of chondrosterin J (**2**).

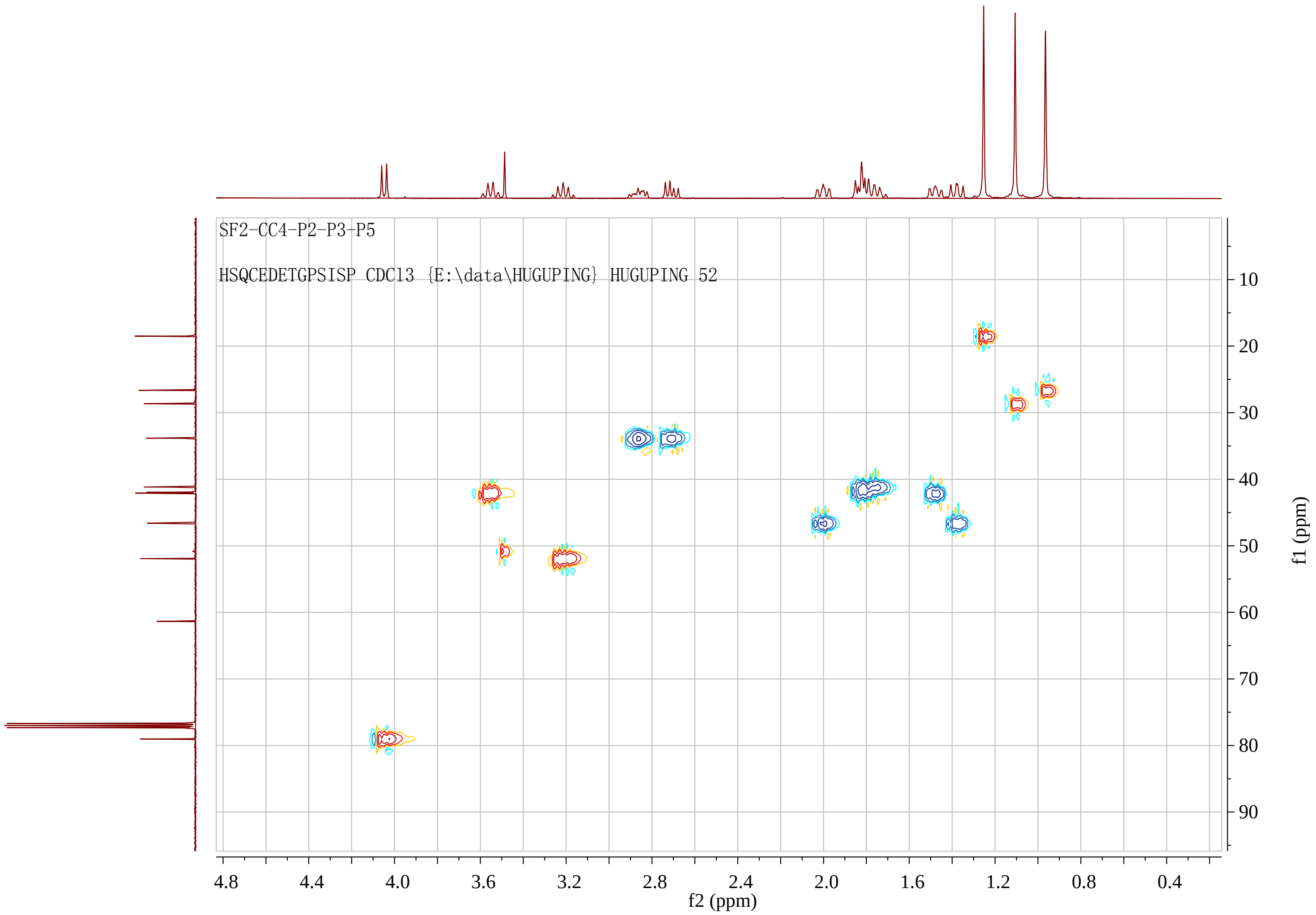

Figure S15. ^{1}H-^{1}H gCOSY spectrum of chondrosterin J (**2**).

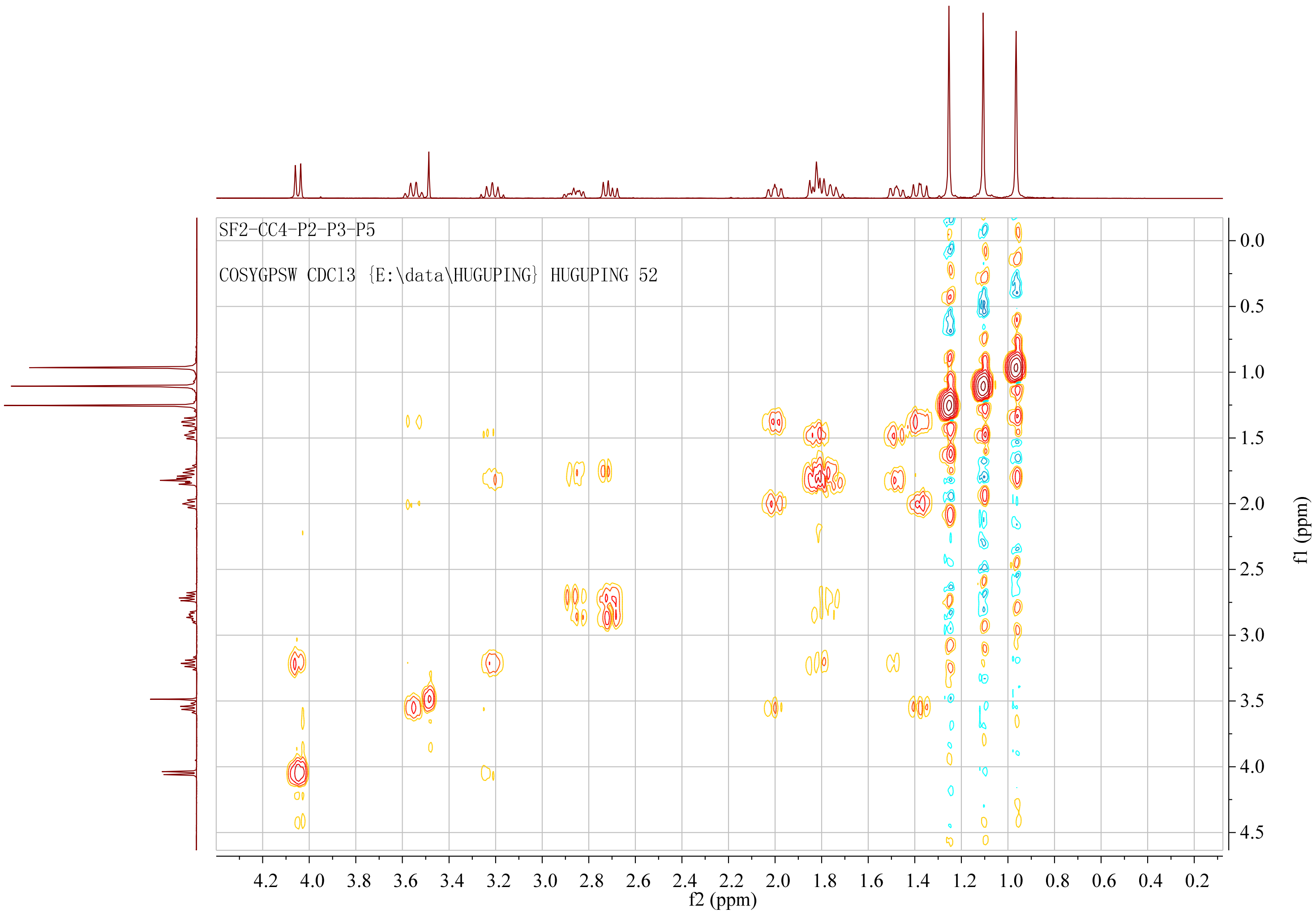

Figure S16. gHMBC spectrum of chondrosterin J (**2**).

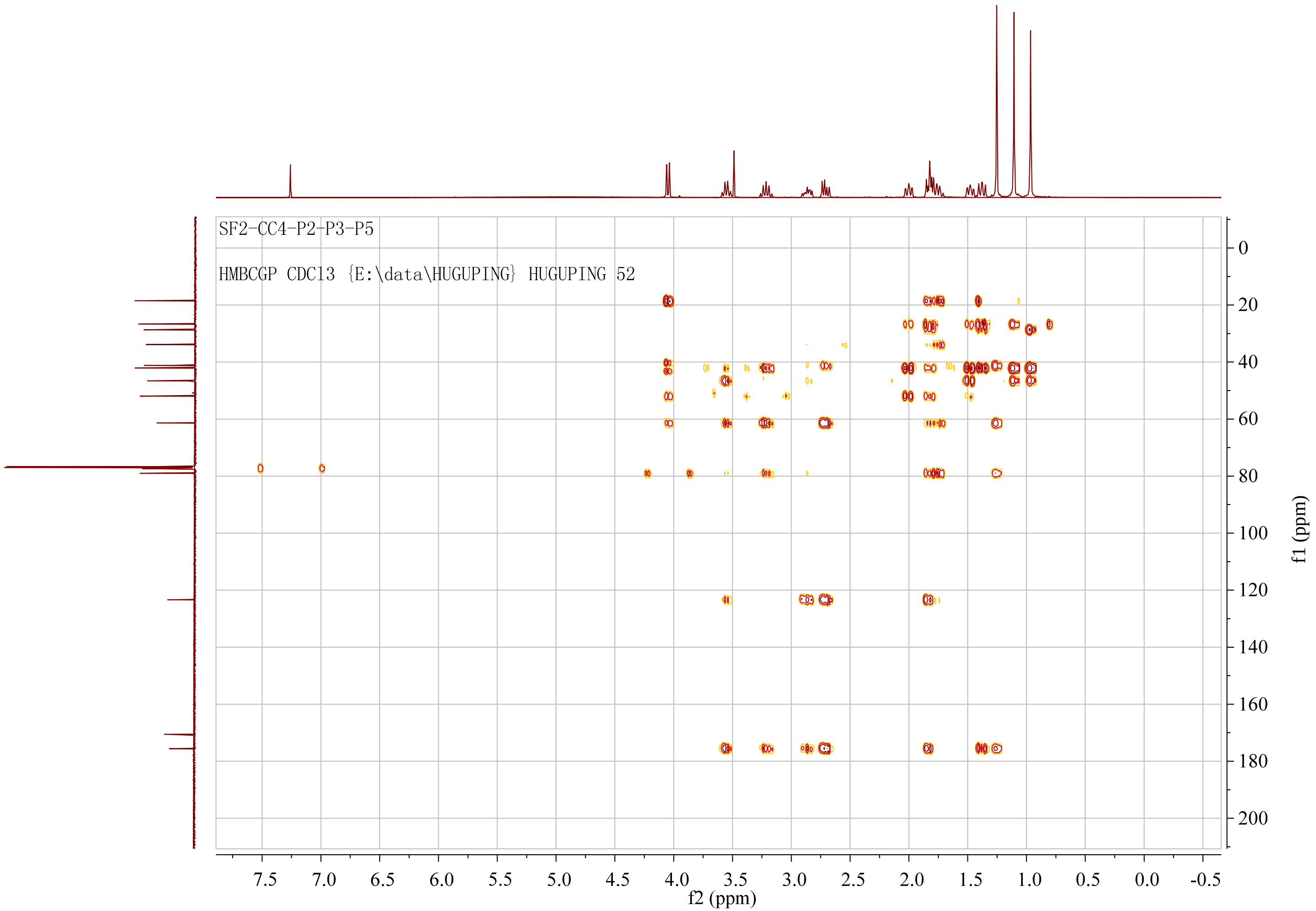

Figure S17. NOESY of chondrosterin J (**2**).

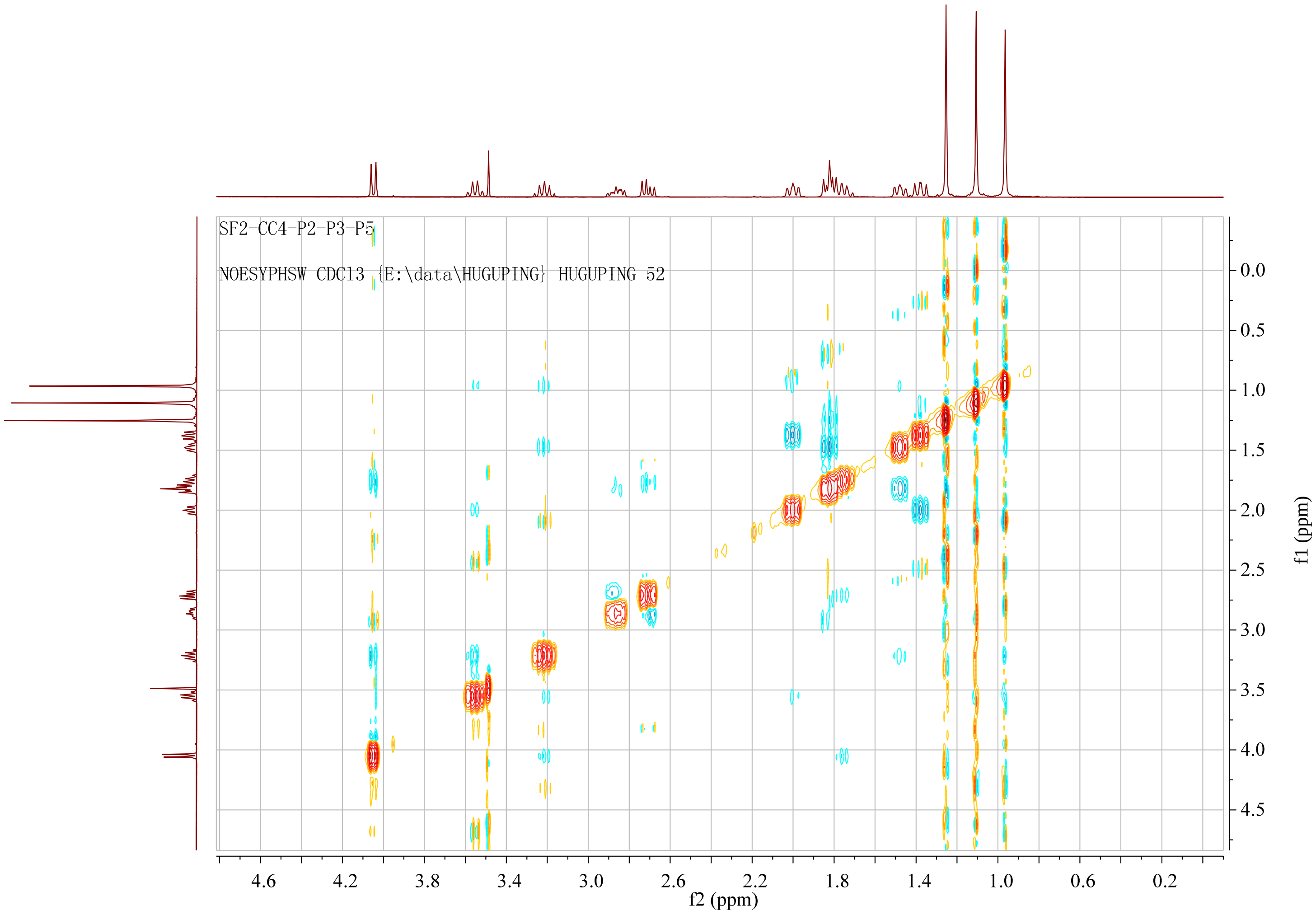

Supplement: Supplementary File 1 — Supplementary Information (PDF, 863 KB) [file marinedrugs-12-00167-s001.pdf]
